# Falls and potential therapeutic interventions among elderly and older adult patients with cancer: a systematic review

**DOI:** 10.4314/ahs.v21i4.34

**Published:** 2021-12

**Authors:** Walid Kamal Abdelbasset, Gopal Nambi, Shereen H Elsayed, Ahmad M Osailan, Marwa M Eid

**Affiliations:** 1 Department of Health and Rehabilitation Sciences, College of Applied Medical Sciences, Prince Sattam bin Abdulaziz University, Al-Kharj, Saudi Arabia; 2 Department of Physical Therapy, Kasr Al-Aini Hospital, Cairo University, Giza, Egypt; 3 Department of Rehabilitation Sciences, Faculty of Health and Rehabilitation Sciences, Princess Nourah bint Abdulrahman University, Riyadh, Saudi Arabia; 4 Department of Physical Therapy, College of Applied Medical Sciences, Taif University, Taif, Saudi Arabia; 5 Department of Physical Therapy for Surgery, Faculty of Physical Therapy, Cairo University, Giza, Egypt

**Keywords:** Cancer, falls, elderly, older adults, risk factors, intervention

## Abstract

**Objectives:**

The aim of this study was to perform a systematic review for previous publications that have assessed the incidence, risk factors, and favorable procedures to prevent and manage falls among cancer survivors of elderly and older adults.

**Materials:**

This systematic review was undertook using PubMed, SCOPUS, Web of Science, Medline, and Cochrane Database of clinical studies and systematic reviews to determine the incidence, risk factors, favorable inpatient and outpatient management, and non-pharmacological interventions for falls among elderly and older adult patients with cancer from 2010 to October, 2020.

**Results:**

After the comprehensive screening, clinical studies, meta-analysis, systematic reviews, and established guidelines were included in this review. Only 5 clinical studies (3 randomized and 2 single-arm studies), 5 systematic reviews, and 6 established guidelines were considered eligible. The five systematic reviews provide risk factors of falls and the 6 guidelines provide assessment & prevention modalities of falls, however, the 6 clinical studies provide the non-pharmacological intervention for falling among cancer survivors. Many factors associated are demonstrated among wide range of elderly individuals. Earlier falls were reliably listed as an important risk factor of falls in the two inpatient and outpatient environments including both general older people and geriatric cancer populations.

**Conclusions:**

This review concludes that the assessment of falls among older individuals with cancer is the most important way for determining who could need additional observation and treatment program. Health professions involving physical therapy and occupational therapy have an important function for promoting health well-being in elderly and older adults with cancer.

## Introduction

Fall is a critical health issue in the elderly and older adult people 1 and is an additional problem for cancer patients because of the severity of the disease and its management.^2^ Number of aged people is continuously increased^3^ and the most number of cancer patients are older adults, cancer care teams countenanced with the dispute of presenting a benefit healthcare for those individuals. Evaluation and treatment of falling is essential because of its severe complications, including functional performance and quality of life among older adults. Fall can be frequently prevented and several threat factors are acquiescent to purposed treatment program such exercise training for muscle strength, balance control, cognitive functions, and medical reviews.^4^ A proper evaluation may detect the causes and risk factors of falling which provide the appropriate modalities for controlling falls and related negative influences.

Cancer is identified an aging disease. The growing number of aged population combined with incidence of cancer may regard a predictable sixty-seven increasing in the prevalence of cancer among older people in 2030.^5^ Regarding the high incidence of cancer among aged people, the threats of falling may be increased and the clinical requirements for preventing falls will be necessitated. Providing great superiority cancer care should be well synchronized among older people.^6^

Accordingly, recent studies should identify the whether the falls risks will be grown among elderly and older adult patients with cancer, and identify the factors associated with falls among those susceptible individuals. Therefore, we conducted a systematic review to assess the rate, risk factors, and proper interventions of falls among elderly and older adult patients with cancer.

## Materials & methods

According to the guidelines and framework of PRISMA checklist^7^, this systematic review was approved by ethics committee of the department of health and rehabilitation sciences at Prince Sattam bin Abdulaziz University (RHPT/019/049). The researchers have explored medical literatures from PubMed, SCOPUS, Web of Science, Medline, and Cochrane Database of Systematic Reviews from 2010 to October, 2020.

Identical provisions and keywords (in differing arrangements) were included in the exploring for concepts of cancer, elderly, older adults, falls, risk factors of falls, prevention of falls, and screening. The search was limited to the English language.

References which indexed in the articles were also utilized to recognize additional researches. Regarding the factors associated with falls among cancer survivors, the search strategy was arranged in accordance with indoor and outdoor environments (despite a number of the guidelines is relevant to both settings) as the risk factors associated with falls are not essentially similar. Guidelines were also sorted according to the inpatient and outpatient setting (although some guidelines were applicable to both) because fall risk factors are not necessarily universal across locations.

Clinical studies were included to detect the non-pharmacological interventions in the treatment of falls among cancer survivors. Regarding consistency, the evidence levels of interferences declared by the strategy were transformed in accordance with the following criteria: 1) systematic reviews of randomized studies, 2) randomized studies/observation studies with impressive impacts, 3) non-randomized cohort studies/prospective studies, and 4) case reports and case-control studies.

## Results

From the comprehensive screening, clinical studies, meta-analysis, systematic reviews, and established guidelines were included in this review. 5 clinical studies (3 randomized and 2 single-arm studies), 5 systematic reviews, and 6 established guidelines were considered eligible. The five systematic reviews provide risk factors of falls and the 6 guidelines provide assessment & prevention modalities of falls, however, the 6 clinical studies provide the non-pharmacological intervention for falling among cancer survivors.

### Risk factors of fall

Five systematic reviews provided the risk factors of falls among elderly and older adults with cancer 2,8–11, many factors associated falls are demonstrated among wide range of elderly individuals. Previous falls were reliably listed as important risk factors of falls in the two inpatient and outpatient environments including both general older people and geriatric cancer populations.^8^–^11^ Whereas the disturbed activity daily livings is provided as a main risk factors in outpatient settings including both general older peopleand geriatric cancer populations^2^,^10^ as detailed in Table 1.

**Table 1:** Risk factors of fall among cancer elderly and older adults

Author, year	Included criteria	Outcomes
**Outpatients**		
Sattar et al., 2020 8	Meta-analysis, systematic reviews, and guidelines which published in English language. Studies on older adults with cancer suffering falls were selected.	According to previous guidelines and systematic reviews, the main risk factors of falls were the previous falls and independent activity daily livings.
Sattar et al., 2016 9	Cross-sectional studies, case-control, qualitative studies, and clinical trials which publish in English language. The age of included samples in these studies was ≥ 60 years. They diagnosed with cancer and were assessed for falls.	According to significant results in two studies (prospective), previous falls is an important factor associated falls among older adults with cancer.
Wildes et al., 2015 2	Clinical trials, prospective and retrospective cohort studies, and case-control studies. All participants suffering cancer or diagnosed with cancer. Falls were assessed as a primary or secondary measure.	According to significant results of one cross-sectional study at least, the risk factors were age, sex, independence of activity daily livings, number of comorbidities, previous falls, white race, marital status, depression status, time up and go score, cognitive dysfunction, using of benzodiazepine and antidepressants, and symptoms related cancer therapy such as chemotherapy, neurotoxic cycles, and pain.
Tinetti et al., 2010 10	Cohort observational studies (prospective) exploring ≥ one risk factor among cancer older adults experiencing falls.	According to the evidence, the independent risk factors were age>80 years, female gender, pain, physical limitations, walking and gait dysfunctions, previous fall, visual dysfunction, muscle weakness, balance deficits, multi-medications, dizziness, depression, diabetes, orthostatic hypotension, arthritis, incontinence, and cognitive dysfunctions.
**Inpatients**		
Sattar et al., 2020 8	Meta-analysis, systematic reviews, and guidelines which published in English language. Studies on older adults with cancer suffering falls were selected.	According to previous guidelines and systematic reviews, the main risk factor of falls was previous falls.
Sattar et al., 2016 9	Cross-sectional studies, case-control, qualitative studies, and clinical trials which publish in English language. The age of included samples in these studies was ≥ 60 years. They diagnosed with cancer and were assessed for falls.	According to significant results in two studies (prospective), cognitive dysfunction is an important factor associated falls among older adults with cancer.
Wildes et al., 2015 2	Clinical trials, prospective and retrospective cohort studies, and case-control studies. All participants suffering cancer or diagnosed with cancer. Falls were assessed as a primary or secondary measure.	According to significant results of one cross-sectional study at least, the risk factors were age, sex, previous fall, physical activity, Using of walk aids, COPD, chronic renal disease, delirium, cognitive dysfunction, using of antidepressants, benzodiazepine, opiates, antipsychotics, and corticosteroids, and symptoms related to cancer treatment including hypotension, pain, anemia, fever, fatigue, and metastases.
Deandrea et al., 2010 11	Prospective studies were selected in order to individuals' number suffering ≥ one fall during the study period, and at minimum eighty percent of the study participants aged ≥ sixty-five years.	Disability, using of walk aids, and falls history are strong indicators for falls in the future.

### Management of fall

Some reviews and guidelines have demonstrated the favorable interventions for falls in the inpatient environments,^12^–^14^ whereas others have identified the favorable modalities to manage falls in the outpatient environments.^4^,^14^–^18^

Inpatient management; as demonstrated in Table 2, previous guidelines 19–21 have recommended assessment of risk factors associated falls after definitive changes of health condition, daily assessment of risk factors, universal exercise for preventing falls,^19^,^22^ and preventing falls by circuit practical approaches.^20^ Also, hospitalized individuals after falling should be considered for obtainable and significant protective management.^19^ Physical exercise training for improving balance and muscle strength, preferably, custom-made to functional performance and ability of the patient is required.^20^ In addition, evaluation and intervention of combined multifactorial treatments such as visual, environmental, supplementations of calcium and vitamin D with exercise training are effective in decreasing fall risk of injuries.^17^ Moreover, patient education and adherence to multifactorial treatment have been recommended.^18^, ^23^

**Table 2:** Guidelines for inpatient management

Agency, year	Management
NICE, 2017 19	Before moving patient when falls during hospitalization, assessing for fractures or spinal cord injuries. Providing the clinical investigations when falling takes place during hospitalization. Home risk and safety evaluation when falls during hospitalization.
RNAO, 2017 20	Assessing the risk of fall risks on hospitalization. General precautions for conducting exercise. Carry out rounds hourly. Before moving patient when falls during hospitalization, assessing for fractures or spinal cord injuries. Providing the clinical investigations when falling takes place during hospitalization. Providing post-falling evaluation and realize modifications when falling takes place during hospitalization. Adherence to exercise training. Achieving multifactorial interventions for preventing falls as a section of the interdisciplinary team. Educating the patient. Modifying the environment. Utilizing of hip protector for preventing hip fractures. Connecting between the risks and plan of treatment to achieve continuing care. Providing the information in proper language and in different forms.
AHRQ, 2013 21	General precautions for conducting exercise. Daily assessment of fall risks. Carry out rounds hourly.

NICE: National Institute for Health and Care Excellence; RNAO: Registered Nurses Association of Ontario; AHRQ: US Agency for Healthcare Research and Quality.

Outpatient management; as detailed in Table 3, previous guidelines 19–21 have provided the following instructions. Firstly, proper evaluation combined with multifactorial treatments provide the definitive factors associated with falls for patients who suffering a highly risk for falling.^19^,^20^,^22^–^24^ Secondly, exercise intervention is highly recommended such as adherence to home exercise or group exercise interventions may improve muscle strength, balance, and walking manner.^4^ Also, yoga exercise has beneficial effects and prevents falling.^18^ Exercise intervention has to be programmed regarding the functional performance and abilities of the individuals.^20^ Moreover, evaluation and intervention of combined multifactorial treatments such as visual, environmental, supplementations of calcium and vitamin D with exercise training are effective in decreasing fall risk of injuries.^17^

**Table 3:** Guidelines for outpatient management

Agency, year	Management
USPSTF, 2018 23	Comprehending evaluation+ personalized multifactorial treatment. Offering exercise intervention to incorporate strength, balance, and gait exercise.
NICE, 2017 19	Regular assessment of falls. Comprehending evaluation+ personalized multifactorial treatment. Referring for balance and strengthening exercise.
RNAO, 2017 20	Regular assessment of falls. Assessment of balance and gait. Comprehending evaluation+ personalized multifactorial treatment. Assessing fall frequency and circumstances. Offering exercise intervention to incorporate strength, balance, and gait exercise. Encouraging dietary therapy to improve bone well-being. Referring to primary care giver and geriatrician. Reviewing medical prescriptions.
NCCN, 2017 24	Regular assessment of falls. Assessment of balance and gait. Comprehending evaluation+ personalized multifactorial treatment. Assessing fall frequency and circumstances. Offering exercise intervention to incorporate strength, balance, and gait exercise. Vitamin D complementation. Referring to primary care giver and geriatrician. Evaluating home safety. Reviewing medical prescriptions.
AGS/BGS, 2010 22	Regular assessment of falls. Assessment of balance and gait. Comprehending evaluation+ personalized multifactorial treatment. Assessing fall frequency and circumstances. Offering exercise intervention to incorporate strength, balance, and gait exercise. Obtaining the history of relevant factors associated with fall. Offering exercise intervention to incorporate strength, balance, and gait exercise. Vitamin D complementation. Evaluating home safety. Reviewing medical prescriptions. Assessing orthostatic hypotension. Assessing feet/feet wear difficulties. Education. Referring to proper physicians/professional teams for additional evaluations and recognize proper managements.

USPSTF: U.S. Preventive Services Task Force; NICE: National Institute for Health and Care Excellence; RNAO: Registered Nurses Association of Ontario; AGS/BGS: American Geriatrics Society BGS.

Other recommendations were provided for preventing falls such as the frequent assessment of falls^19^,^22^,^24^, assessment of balance and gait^22^,^24^, and nutritional therapy with supplementation of vitamin D. 19,20,22–24 In addition, regular evaluation of home safety,^22^,^24^ behavioral modifications, review of medical prescriptions, particularly high risky drugs contributing to fall such as hypotensive and antipsychotic medications 22 are greatly recommended for preventing falls. Moreover, patient and caregiver education in combining with other recommendations plays an important role for management of falls in outpatient settings.^20^,^22^

### Non-pharmacologic interventions

Nutrition and physical exercise therapy showed an improvement in cancer care field.^25^ A preliminary prospective single-arm study concluded that 8-week multimodal exercise intervention has a potential therapeutic value in improving balance, mobility, and quality of life, and thereby reducing the incidence of fall among cancer survivors with chemotherapy.^26^ It was also documented that 1-year phoned physical activity including aerobic, strength, balance, and flexibility exercise may prevent the reduction in physical performance among older adults with cancer.^27^ Therapeutic modified Tai Ji balance exercise for 24 weeks showed a considerable reduction in the incidence of falling among older adults exposed to a great risk of falling.^28^ 4-week sensor-based exercise program also demonstrated an improvement in body balance and postural control in older cancer survivors with chemotherapy.^29^ Furthermore, Lee et al., 2016 found that 6-week community-based multimodal physical activity intervention including aerobic, strength, and balance exercise training may improve physical functions-related falling risk factors among cancer survivors.^30^ Additionally, It was reported that resisted exercise safely achieves an improvement in upper and lower body muscle strength, and therefore lowering the risk factors for falling and disabilities.^31^ Table 4 shows exercise interventions for falls among elderly and older adults with cancer.

**Table 4:** Exercise interventions for falls among elderly and older adults with cancer

Author, year	Modality of treatment	Outcome measures	Main findings
McCrary et al., 2019 26	8-week exercise intervention (3- weekly sessions).	Objective and patient-reported, standing and dynamic balance, mobility, quality of life, and sensory and motor nerve excitability.	Objective and patient-reported, dynamic balance, standing balance in eyes open conditions, mobility and quality of life were improved.
Arrieta et al., 2019 27	1-year phoned physical activity, twice a week (strength, balance, flexibility, and aerobic exercises).	Short physical performance battery (SPPB) and cognitive assessments.	Reduced physical performance decline 2-year post- intervention.
Schwenk et al., 2016 29	4 weeks (2 sessions a week, 45 min each) interactive game-based balance training including repetitive weight shifting and virtual obstacle crossing tasks.	Sway of hip, ankle, and centre of mass in both mediolateral and anteroposterior directions during balance test, standing in feet-closed with opened eyes and closed eyes, gait performance, and fear of falls.	Reduction in hip, ankle, and centre of mass sways, improvement in standing in feet-closed position with opened eyes, except anteroposterior centre of mass sway, and in semitandem position except ankle sway.
Lee et al., 2016 30	6-week multimodal physical activity (aerobic, strengthening, and balance exercise).	4-meter walk, chair stand, one-leg stance, tandem walk, and dynamic muscular endurance tests.	Improved physical functionrelated risk factors for falls, and lowered risk for falls based on specific physical functionrelated falling.
Kerri et al. 2012 31	1-year resistance + impact training exercise (1 hr supervised training twice a week and 1 hr home-based exercise a week).	4 m usual walk speed, 1 rep. max. leg and bench press strength, self-report physical function, handgrip strength, 5 chair stand, stance-test durations and fatigue.	Improved maximal muscle strength. Improved lower and upper body strengths, thereby reducing the incidence of falls.

## Discussion

We conducted the current systematic review to assess the risk factors and proper interventions for falls among elderly and older adult patients with cancer. The findings showed that the falls history is the commonest risk factor for falling among elderly and older adults suffering cancer whether inpatient or outpatient settings. Consequently, elderly individuals have to be assessed for recent falls when happened. Despite the variations of interventions were recommended, assessing falls each medical visit, particularly in the outpatient or outdoor settings as the aged people are commonly suffering from quick functional impairments related to the development of cancer disease.

Twenty percent of elderly individuals are experiencing disturbance in activity daily livings when exposing to chemotherapy.^32^ For that reason, the risk factors of falls are not fixed, but it may be changed severely at the treatment period of cancer disease.^2^ Various assessment methods were performed by health professions involving functional performance of activity daily livings and realizing the proper environmental modification to promote home safety.^33^ Hence, physical and occupational therapy may have an effective role in the interdisciplinary team for handling geriatrics with disturbed activity daily livings.

The several strategies of falls management were highly recommended by the reviewed guidelines. Combination between exercise intervention and the multifactorial evaluation and treatment also is highly commended for preventing and managing falls among elderly people, particularly experiencing cancer.^19^,^20^,^22^–^24^ Exercise intervention and physical activity training may reduce falls risks and injuries in elderly individuals through controlling weight and preserving healthy conditions of musculoskeletal system,^34^ also improve balance, mobility, and gait parameters.^35^

Physical therapy assessment has a key role for proper evaluation of the musculoskeletal system, especially physiotherapists who have expertise in assessment of falls and have the sufficient skills for creating safe exercise programs for preventing falls among elderly individuals.^36^ Utilizing specific strategy for exercise intervention, physiotherapist could adjust the proposed program for elderly people according to the assessment outcomes of the physical performance.^37^ Moreover, outdoor activities including walking or gait training are likely sufficient and easy method for conducting exercise intervention which improve mobility, muscle strength, flexibility, balance, gait, and reduce the risks of falls.^38^

## Conclusions

Fall among elderly and older adult patients with cancer is growing as the number of aged people is continuously increased. The current review provides the common risk factors of falls and the efficient modalities for preventing falls in those individuals according to the published systematic reviews and the created guidelines.

This review concludes that the assessment of falls among older individuals with cancer is the most important way for determining who could need additional observation and treatment program. Health professions involving physical therapy, occupational therapy, and exercise intervention have an important function for promoting health well-being in elderly and older adults with cancer. However, future studies examining the different effects of tailored technology-based training and traditional exercise intervention are required to achieve the most desired benefits in preventing falls among elderly and older adults.
